# First genetic linkage map of *Taraxacum koksaghyz* Rodin based on AFLP, SSR, COS and EST-SSR markers

**DOI:** 10.1038/srep31031

**Published:** 2016-08-04

**Authors:** Marina Arias, Monica Hernandez, Naroa Remondegui, Koen Huvenaars, Peter van Dijk, Enrique Ritter

**Affiliations:** 1 Neiker Tecnalia (Granja Modelo de Arkaute), Ctra. N1-Km 355, Arcaute (Alava) CP: 01192, Spain; 2 KeyGene, Agro Business Park 90, Wageningen, 6708 PW, The Netherlands

## Abstract

*Taraxacum koksaghyz* Rodin (TKS) has been studied in many occasions as a possible alternative source for natural rubber production of good quality and for inulin production. Some tire companies are already testing TKS tire prototypes. There are also many investigations on the production of bio-fuels from inulin and inulin applications for health improvement and in the food industry. A limited amount of genomic resources exist for TKS and particularly no genetic linkage map is available in this species. We have constructed the first TKS genetic linkage map based on AFLP, COS, SSR and EST-SSR markers. The integrated linkage map with eight linkage groups (LG), representing the eight chromosomes of Russian dandelion, has 185 individual AFLP markers from parent 1, 188 individual AFLP markers from parent 2, 75 common AFLP markers and 6 COS, 1 SSR and 63 EST-SSR loci. Blasting the EST-SSR sequences against known sequences from lettuce allowed a partial alignment of our TKS map with a lettuce map. Blast searches against plant gene databases revealed some homologies with useful genes for downstream applications in the future.

Over 12, 1 million tons of natural rubber (cis-1,4-polyisoprene) were consumed in the World in 2014. Sold in Europe at 1811 US$ per ton, natural rubber moved many billions that year^1^. Today, all natural rubber is harvested from the tropical rubber tree, *Hevea brasiliensis*. However, rubber tree plantations are vulnerable to pathogens, climate change, shortage of skilled tappers, replacement by more profitable oil palm, etc. Moreover, rubber production from rubber tree is not flexible since it takes 7–8 years before a rubber tree can be tapped for the first time. These factors drive the search for alternative or additional natural rubber crops.

*Taraxacum koksaghyz* Rodin (TKS) is a perennial rosette-forming plant species, belonging to the Asteracae family. TKS latex contains high quality natural rubber with an average molecular weight of 2180 KD, comparable to that one coming from *Hevea brasiliensis*^2^. TKS originates from SE Kazakhstan near the border with China, where it grows in high valleys of the Tien Shan Mountains at 1700–2000 m altitude^3^. Most rubber is found in the tap root (on average 5% on dry weight basis in wild germplasm). In addition to rubber TKS roots contain inulin (25–40% on dry weight basis in wild germplasm^2^). This poly-fructan is a valuable by-product that can be used in food as dietary fibre or in non-food as building blocks for bio plastics or bio fuels. Cultivation and harvesting of TKS can be mechanized. Therefore, TKS has the potential of an annual rubber crop for the temperate regions.

Companies such as Apollo Vredestein and Continental are already testing tires built from TKS rubber and close to commercialization of these new products. Continental expects serial production within ten years^4^,^5^. Although TKS was grown as a rubber crop in the Soviet Union until the mid-1950s, no improved germplasm is existing anymore^3^. At the moment only wild germplasm is available. In order to achieve an improvement of this species for covering the needs of TKS rubber production, a breeding program is needed urgently. In contrast to most dandelion species, TKS is not a polyploid apomictic species, but a diploid species (2n = 16) that reproduces sexually by outcrossing. TKS has a genome size of 1.45 pg/1C (1420 Mb)^3^. The moderate genome size and the short life cycle (4 month in the greenhouse; own observations) makes TKS highly amenable for plant breeding.

Molecular markers play a crucial role for efficient developing of improved plant crop germplasm. They have provided a major contribution to the genetic knowledge of many cultivated plant species. In addition to their basic importance for genetic and evolutionary studies, molecular markers are useful to construct linkage maps and to localize monogenic and polygenic traits which allow the efficient introgression and selection of individuals with specific characteristics. Molecular techniques to assist breeding are applied nowadays to all major crops. They offer several advantages which are difficult to achieve using only classical breeding methods and speed up the whole selection process. Particularly, marker-assisted selection allows identifying appropriate genotypes already in seedling stage. Marker-assisted selection can also substitute laborious, expensive and time consuming processes for evaluating particular characters in progeny genotypes. They also play a crucial role in the identification, isolation and cloning of plant genes of interest^6^. However, only a limited amount of genomic resources exist currently in TKS and particularly no linkage map is available.

Different Polymerase Chain Reaction (PCR) marker types can be used for the construction of linkage maps. The Amplified Fragment Length Polymorphism (AFLP) technique is a multi-locus (fingerprinting) technique which is PCR based^7^,^8^. It can be used in order to obtain quickly a skeleton map with full genome coverage and for closing gaps within linkage maps^9^.

On the other hand co-dominant markers such as Simple Sequence Repeats (SSR) or Conserved Orthologues Set (COS) markers are useful for aligning different linkage maps in the same species, since they map to identical genomic locations in different genetic backgrounds or they can be even used for synteny studies between different species^10^,^11^. Particularly SSR are widely used as genetic markers, because they are co-dominant, multi allelic, easily scored and highly polymorphic^12^. Sequence Tagged Sites (STS) and (Expressed Sequence Tag) ESTs are unique sequences obtained by cDNA sequencing^13^. ESTs provide a robust sequence resource that can be exploited for gene discovery, genome annotation and comparative genomics^14^.

In order to obtain the first genetic map of TKS cheap techniques as AFLP, SSR and COS markers were selected. In addition EST-SSRs or genic SSR markers are relatively easy and cheap to produce, since they are byproducts of the sequence data from genes or ESTs that are publicly available. Their generation is limited to those species that are close relatives and for which a sufficient large number of ESTs are available. Another advantage over genomic SSRs is that they are present in expressed regions of the genome. Since limited genomic sequence data are available for many plant species, EST databases have been screened for the development of more universal EST-SSRs^15^.

It has been proven that linkage groups (LG) are conserved to a certain degree between closely related species and even to a less degree between species of the same genera or family^13^. Conserved marker order is referred to as ‘synteny’^16^. The term synteny is used in genetics to indicate the presence of two or more loci from different species on the same chromosome in each species. Today, the concept of synteny has been also expanded to address questions of sequence homology of genes and stretches of homologous chromosomes^17^. Synteny studies by comparative mapping may assist in the construction of new linkage maps (or localized maps of specific genomic regions) and in predicting the locations of (Quantitative Trait Loci) QTLs in different mapping populations^18^. Previous existing linkage maps may provide an indication of which markers are polymorphic, on which LGs they are located and the order of markers within a LG. Furthermore, comparative mapping may reveal evolutionary relationships between taxa^16^.

The use of sequenced markers which have a concrete biological meaning for linkage map construction allows obtaining a functional map. In this context a major step to make sequence information publicly available for large-scale analysis was the formation of GenBank and its counterpart, the European Molecular Biology data library in 1982. As more and more sequences are deposited, these and other databases are becoming increasingly useful, because there is an ever greater chance of finding sequence similarity for a newly sequenced gene^17^.

The aim of this study was to establish a high-density linkage map of TKS based on AFLP, COS, STS, SSR and EST-SSR markers and to exploit sequenced markers by homology searches to detect useful genes and for synteny studies with related species.

## Results

### Marker Polymorphisms

In this study a high density linkage map has been developed. The parents in the cross were highly polymorphic for the AFLP markers, consistent with the high amount of AFLP variation observed in a previous Genetic Diversity Analysis of TKS germplasm collected in 2008^5^. The 20 evaluated AFLP primer combinations (PCs) generated a total of 763 polymorphic fragments (without duplicated common fragments), varying from 30 to 69 between primer combinations. A total of 275 bands were specific for the father, 284 descended from the mother and 206 were present on both parents.

From the additional 196 evaluated EST-SSR, STS, SSR and COS markers only 69 were found to be useful. They produced between 1 and 6 segregating bands on the gels. The other markers were either monomorphic, did not amplify or produced unclear, smeary bands on the gels, even after adjusting PCR conditions in many cases. From the 128 evaluated EST-SSR or TC markers (given name to analyzed EST-SSR), 58 revealed segregating polymorphisms generating a total of 147 bands (See Supplementary Table S1b). For the 10 evaluated STS markers only from three markers, a total of five segregating bands were obtained. From the 31 evaluated COS markers only 7 published by Chapman *et al*.^19^ and none from the five COS from CGDPB^20^ were found to be useful. The seven polymorphic COS markers produced a total of 11 segregating bands. Only one of the SSR published by Simko^21^ of the 27 analyzed SSR primer combinations produced one segregating band. None of the 17 sunflower SSRs^22^ was useful (See Supplementary Table S1a,b). In this way additional 69 PCs derived from COS, SSR, STS and EST-SSR markers were available for linkage mapping with a total of 164 segregating amplification products, summing up to 89 PCs with 927 bands including AFLP markers.

### Linkage Maps

#### Parental Maps

Using the described methodology, initially individual parental linkage maps were produced in the TKS mapping population. In both cases and since the maps were sufficiently saturated, eight linkage groups were obtained corresponding to the eight chromosomes of the haploid TKS genome. The characteristics of the individual parental maps are shown in Tables 1a,b.

A total of 295 markers could be integrated into the Parental 1 “low rubber producer” (P1-L) map. Individual linkage groups of P1-L contain between 30 and 46 markers each. On average a linkage group is composed of 23.1 P1-specific AFLP markers, 8.8 common AFLP markers and 5.0 COS/SSR/EST-SSR markers, summing up to 36.9 markers per linkage group. Individual linkage groups of P1-L vary between 65.4 and 120.7 cM in length, the total P1 map length was 789.4 cM with an average linkage group length of 98.7 cM (Table 1a).

A total of 297 markers could be integrated into the Parental 2 “high rubber producer” (P2-H) map. Individual linkage groups of P2-H contain between 23 and 50 markers each. On average a linkage group is composed of 23.5 P2-specific AFLP markers, 8.4 common AFLP markers and 5.3 COS/SSR markers, summing up to 37.1 markers per linkage group. Individual linkage groups of P2 vary between 80.4 and 123.1 cM in length, total map length of P2 is 820.1 cM, with an average LG length of 102.5 cM (Table 1b).

#### Integrated Map

The characteristics of the final integrated map are shown in Table 1c. For the construction of the integrated map as described above a total of 62 common AFLP anchor fragments and 32 COS/SSR/EST-SSR anchor markers were available. The integrated map contains 185 individual AFLP markers from P1, 188 individual AFLP markers from P2, 75 common AFLP markers and 70 COS/SSR/EST-SSR loci, leading to a total of 518 markers in the map.

The map has a length of 894.1 cM with an average linkage group length of 111.8 cM. Individual linkage groups vary between 88.9 and 135.9 cM in length and contain between 46 and 84 markers each. On average a linkage group is composed of 23.1 P1-specific AFLP markers, 23.5 P2-specific markers, 9.4 common AFLP markers and 8.8 COS/SSR/EST-SSR markers, summing up to 64.8 markers per linkage group.

**Figure 1:**
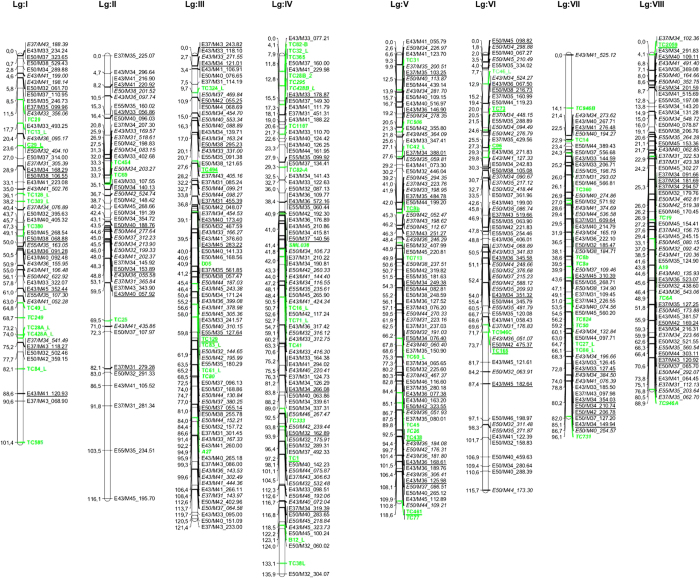
Integrated Linkage Map of *Taraxacum koksaghyz* Rodin. Common fragments mainly used as anchor points are underlined. AFLP fragments are indicated with their Fragment names. Markers from P2 are indicated in Italics. COS/SSR/EST-SSR markers are indicated in “bold light-green”.

The integrated TKS map is visualized in Fig. 1. In addition, a total of 208 so-called RF0 markers (i.e.: markers which are linked with a recombination frequency (RF) equal to zero to other mapped markers) were mapped, which are not displayed in Fig. 1. Furthermore, 47 so-called associated markers were determined. These markers do not fit precisely in the existing framework maps (probably due to scoring errors), but they show reduced RF values (<10 cM) with other mapped markers and therefore they are “associated” to them. Therefore, the actual marker number in the final integrated map of this cross including RF0 and associated fragments is 773 with an average of 96.6 markers per linkage group. A total of 62 (mainly distorted) AFLP Fragments could not be integrated into the linkage map.

### Mapping of COS, EST-SSR and SSR Markers

A total of 33.2% of the assayed COS and EST-SSR and SSR markers could be perfectly integrated in the genetic map (65 of 196 PCs in total); 6 COS, 1 SSR and 58 EST-SSRs were mapped to 70 loci in the integrated map and to 60 loci in both individual maps (See Supplementary Table S1a).

Depending on the origin of the primer combination the mapping success changed considerably. The highest success was achieved with EST-SSR primers of *T. officinale*; where 58 out of 128 could be mapped (45.3%). COS markers from lettuce and sunflower and SSRs from lettuce, achieved a mapping success of 23.1% and 10% respectively. None of the lettuce COS markers from the *Compositae* Genome Project Data Base (CGPDB), STS from chicory and SSRs from sunflower could be mapped (See Supplementary Table S1b).

### Synteny study

The information provided by Keygene about the locations of lettuce genes homologous to TC sequences on the lettuce map, allowed us to perform a partial alignment of both, TKS and lettuce maps which is shown in Table 2. In some cases a concrete lettuce marker was not identified, but homologies with a lettuce scaffold were established and the location of this scaffold on the lettuce map was known. TC markers of all TKS linkage groups could be mapped to all lettuce linkage groups.

In the case of TKS linkage group 3 all TKS homologs mapped to the same linkage group LG 4 in lettuce. However, in most cases EST-SSR markers from the same TKS LG mapped to different LGs of the lettuce map. This could be due to different genomic arrangements in both species, or in part also due to multi locus markers, as observed during linkage map construction in TKS. In some cases the corresponding LGs of the TKS map might be assigned to LGs of the lettuce map, based on a majority principle that means where most of the markers are located. In this sense TKS LG6 could correspond to lettuce LG3 and TKS LG7 could correspond to lettuce LG1. Considering the higher number of chromosomes in lettuce, LG5 might be associated to a combination of lettuce linkage groups 5 and 6.

### Functional genomics

**Figure 2:**
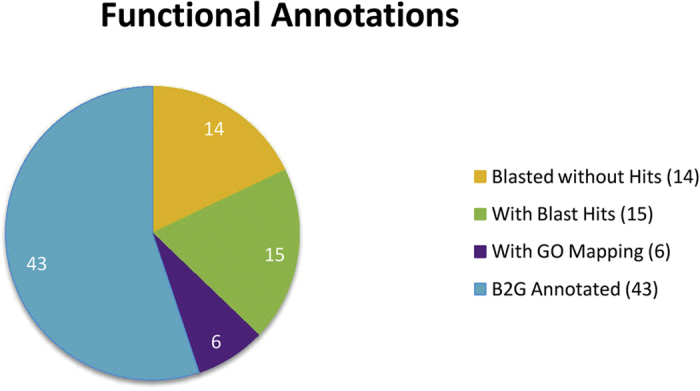
Summary of Blast2GO functional annotation results obtained for TC sequences. Blasted without Hits: number of sequences without Blast hits at the given threshold (e-value < 10^−10^); With Blast Hits: number of sequences with Blast hits at the given threshold (e-value < 10^−10^); With GO Mapping: Number of sequences that mapped to the Blast2GO database; B2G Annotated: Number of sequences that did retrieve one or more GO annotations from the Blast2GO database.

The types of Blast2GO functional annotations of TC sequences are summarized in Fig. 2. The homology search of TC sequences using Blast2Go revealed GO annotations for 43 out of 78 sequences (55.1%). For 18% of the sequences no Blast hit was obtained (14 out of 78), 19.2% (15 out of 78) got Blast hits at the given threshold and 7.7% (6 of 78) could be mapped to the Blast2GO plant gene database (Fig. 2).

**Figure 3:**
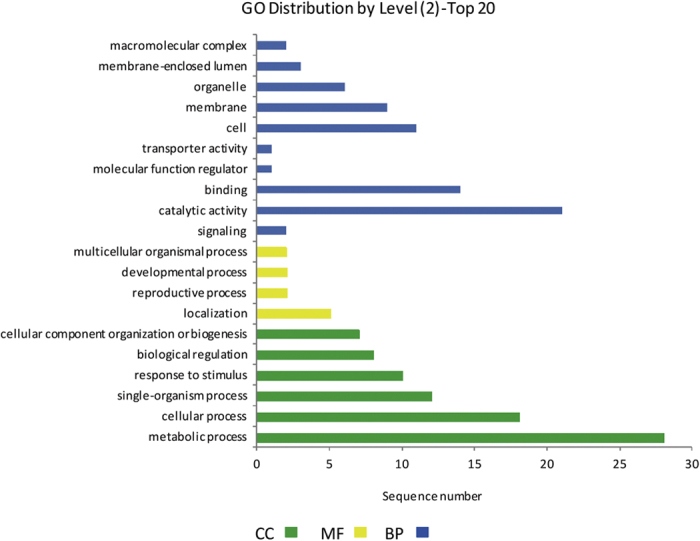
GO distribution for level 2 of TC sequences obtained by Blast2GO. CC: Cellular Component; MF: Molecular Function; BP: Biological Process.

The distribution of GO annotation terms at ontology level equal to 2 is shown in Fig. 3. A total of 26% of the annotations had diverse metabolic process functions.

The Blast2Go analyses of the TC sequences revealed by chance some interesting homologies with known genes which are summarized in Supplementary Table S2. For example, TC 77 shows a high homology with a pyridoxal biosynthetic protein (97% similarity) which is related to stress response. TC249 matches a coatomer subunit zeta-1 of a protein with 95.7% similarity and the gene ontology indicates relationships to a heat response, high light intensity and endoplasmatic reticulum stress (See Supplementary Table S2). The same holds true for the TC428 sequence. TC303 shows a somewhat lower but significant homology (similarity 75.3%, e-value 3.0E-61) with a glycerol-3-phosphate acyltransferase and the Gene Ontology (GO) relates this sequence with the isoprenoid biosynthetic process. In the mapping process TC49 got a hit with the accession gi|496405813|gb|AGL44368.1|Rop5 [*Hevea brasiliensis*] with an e-value of 1,66E-119 (results not shown). TC61 (A, B, C), TC77, TC129 and TC324 have homologies with genes involved in carbohydrate metabolic processes.

## Discussion

We have constructed an integrated, high density map of *Taraxacum koksaghyz* Rodin which contains over 500 markers on the eight linkage groups. Both parents were highly heterozygous, as expected for germplasm of an obligate outcrosser.

The normalization of the AFLP gel images by Software was very useful (KeyGene™ Quantar Suite), since it allowed increasing the information of common markers by distinguishing between homo and heterozygous progeny genotypes. We found the most of the duplicated common markers as described above mapped to identical locations on the individual parental TKS maps and provided many additional anchor points for integrating these maps efficiently.

Numerous co-dominant COS, STS, SSR and EST-SSR markers derived from different *Asteraceae* species were evaluated, but their mapping success was quite variable depending on the origin. The markers from the most closely related species of the same genus were the most efficient markers, as can be seen for the mapping success of EST-SSR markers derived from *Taraxacum officinale*. Fulton *et al*. developed in 2002^11^ conserved ortholog set markers for higher plants. The authors claim that the identified COS markers could be used in a wide variety of plant genomes and that they could facilitate mapping in plant genomes that lack gene or EST databases. These COS markers could be tested, in the future, in our mapping population. If they are suitable for TKS, they could be used for comparative genomics of the TKS genome with those of other higher plants of interest.

The use of markers common to different maps, or markers for which the underlying sequence is available, is useful for linkage map construction, since they allow potentially performing synteny studies by comparative mapping and integration of markers and QTLs from other maps. Since our TKS map is the first that has been published in this species, there is no possibility to compare it to other TKS maps. Therefore, we intended a comparative study with other species of the same family, such as lettuce. Synteny studies can help us to get new sources of heterologous probes for saturating regions including genes of interest^23^ and can help predicting the position of orthologous genes of agronomic interest in related species^24^. Based on our results we can potentially associate homologous linkage groups between TKS and lettuce maps, or at least part of them. However, many additional markers from the TKS and/or lettuce map should be evaluated, in order to improve the alignment or detect concrete structural differences such as inversions or translocations. Even if a full alignment cannot be achieved, the establishment of concrete exploitable syntenic blocks should be possible, as shown previously by Gebhardt *et al*.^25^ who performed comparative mapping even between the more distant species potato and Arabidopsis.

The obtained TKS map sets the basis to establish a functional reference map of the TKS genome by integrating in the future QTLs for quantitative characters such as growth, biomass production, rubber and inulin contents, rubber concentration and quality and other traits of interest after trait recording in the mapping population and QTL analyses. Also the integration of markers for candidate genes from the rubber biosynthesis pathway is foreseen after developing appropriate primers for them. Markers which are co-located with a mapped QTL might explain the potential influence of the underlying candidate gene.

The use of markers with underlying sequences represents another option to increase the functionality of a map. These sequences can be blasted against plant gene databases, sequenced markers from other maps or sequence scaffolds from other species, in order to integrate concrete genes into the map. Some interesting homologies were detected by chance when blasting the EST-SSR sequences that were used for the construction of the TKS genetic linkage map against plant gene databases using Blast2GO. These will be useful for explaining potential QTLs, if they are co-located with QTLs for relevant traits in future studies. For example, TC77, TC249 and TC428 were found to be related with stress responses. Sometimes also rubber formation seems to be triggered by some kind of stress suffered by the plant and particularly in the case of TC77 it matches with a pyridoxal biosynthetic protein. A probable pyridoxal 5-phosphate synthase subunit PDX 1 was also characterized in *Hevea brasiliensis* and its full description is available at the UniProt web site^26^.

TC303 seems to be involved in an isoprenoid biosynthetic process and therefore could be directly related with rubber formation and rubber traits. TC49 obtained a Blast hit with a Rop5 gene from *Hevea brasiliensis* (result not shown derived from mapping process with Blast2Go Pro). Qin *et al*.^27^ explored the characteristics and possible functions of ROP genes which are primarily expressed in laticifers of the rubber tree (*Hevea brasiliensis*). This work represented a first step for the determination of their involvement in latex flow and regeneration, laticifer formation and tapping panel dryness (TPD). This protein could have a similar function in TKS. Also the mapped TC61, TC77, TC129 and TC324 markers which were found to be related with carbohydrate metabolic processes could be potentially related to inulin formation and contents. Further mapping of additional TC markers and future research is needed to search for candidate genes influencing important traits by applying co-location analyses with QTL, expression analyses or other molecular methods.

The amino acid sequence of the rubber elongation factor (REF) associated with rubber particles in *Hevea brasiliensis* is known since the publication of Dennis *et al*. in 1989^28^. In the same year, Light and Dennis^29^ described REF, characterized its role in rubber biosynthesis and purified a prenyltransferase that elongates cis-polyisoprene rubber from latex of *Hevea brasiliensis*. Rubber biosynthetic genes from TKS were cloned and characterized by Schmidt *et al*. in 2010^30^. All this available information can be used for developing molecular markers related to rubber formation in TKS. If they are polymorphic, they can be mapped in our population and their influences on rubber traits could be validated by co-location analyses with QTLs in the future. A similar approach was adopted earlier by Menendez *et al*.^31^ in potato, who associated QTLs for reducing sugar metabolism with relevant enzyme markers from the corresponding metabolic pathway.

Nevin Young wrote in 1994^18^, “Genome mapping is directed towards a comprehensive genetic map covering all chromosomes evenly. This is essential for effective marker-assisted breeding, QTL mapping, and chromosome characterization.” Our present TKS map is though prepared for these kinds of studies.

## Materials and Methods

### Mapping population

In spring 2009 a controlled cross between a TKS genotype with low rubber production (TKS 237-2; P1-L)^5^ and a genotype with high rubber production (TKS 194-1; P2-L)^5^ was performed by KeyGene (Wageningen, The Netherlands) in order to generate a highly variable mapping population. For this purpose a previous vernalization period of 5 weeks at 6 °C was applied to both parents with the aim to induce and synchronize flowering. Both genotypes were raised from seeds collected in the wild^5^,^32^. In June, the seeds from the cross were sown in the greenhouse. Out of 119 grown progeny genotypes, 94 were used as mapping population.

### DNA samples

Genomic DNA was isolated by KeyGene from 2 cm^2^ of fresh and young leaves of all progeny genotypes and parental plants, according to the hexadecyl-trimethyl-ammonium-bromide (CTAB) procedure^33^. These DNA samples were used for AFLP analyses by KeyGene and DNA aliquots of each sample as well as leaf samples were sent to NEIKER for additional DNA extractions and marker analyses.

The quality of the received DNA samples were checked by horizontal electrophoresis in 1% agarose gels in Tris-acetate-EDTA (TAE) buffer and visualization by Ethidium bromide staining. DNA quantification was done using a NanoDrop 2000 Spectrophotometer (Thermo Scientific, USA).

### Molecular Markers

AFLP fingerprinting^6^ was performed by KeyGene for the parents and 94 F1 plants according to the protocol of Vuylsteke *et al*.^34^.

To prepare the AFLP templates 250 ng of DNA per sample was used for the restriction-ligation reactions.

A total of 20 *Eco*RI/*Mse*I primer combinations were analizad: E37/M31, E37/M34, E37/M35, E37/M43, E43/M33, E43/M34, E43/M36, E43/M40, E43/M41, E43/M45, E50/M32, E50/M34, E50/M37, E50/M38, E50/M40, E50/M42, E50/M44, E50/M45, E50/M46, E55/M35.

AFLP Fragments were separated on 4.5% denaturing polyacrylamide gels. AFLPs gels were scored co-dominantly, based on normalized fragment intensity over the entire gel. The intensity of the fragments was quantified using AFLP-Quantar^®^ software. In addition, gels were scored visually for presence and absence of fragments.

Additional, 196 molecular markers were evaluated in the mapping population by NEIKER which were from different origins (See Supplementary Table S1a,b). A total of 26 COS markers descending from EST markers of lettuce and sunflower were analyzed^19^, as well as five COS markers derived from ESTs of *Lactuca sativa* and *L. serriola* from the Compositae Genome Project Data Base (CGPDB)^20^, 10 SSRs derived from ESTs of *L. sativa* and *L. serriola*^21^, 17 SSRs from ESTs of sunflower published by Tang *et al*.^22^ and 10 STS markers identified in ESTs of *Cichorium intybus*^35^.

In addition, around 41000 ESTs from a *Taraxacum officinale* library^20^ (common Dandelion) were used for SSR mining and the design of adequate primers. This service was provided by Clemson University (South Carolina, USA)^36^ using primer 3^37^ with default settings. The service delivered a large amount of SSR, their characteristics and appropriate primers. A total of 128 of these primer pairs were evaluated in our mapping population (See Supplementary Table S1a,b).

All primers used for mapping were synthesized by MWG Biotech AG (Ebersberg, Germany). Forward primer were labeled with an infrared dye (IRD800 or IRD700; LI-COR, Lincoln, Nebraska, USA). Primus 96 or Primus 384 plus thermo cyclers (MWG Biotech AG, Ebersberg, Germany) were used for PCR reactions. Recombinant Taq DNA Polymerase (Invitrogen Inc. Barcelona, Spain) was used for COS markers and DFS-Taq DNA Polymerase (Bioron, Spain) for SSR and EST-SSR markers in the PCR reactions. The annealing temperature suggested by the primer manufacturer was used. PCR reactions were performed according to Sefe *et al*.^38^.

Amplification products were mixed with 7 μl of formamide loading buffer (98% v/v formamide, Ethylenediaminetetraacetic acid (EDTA) 10 mM, pH 8.0, bromophenol blue 0.1% p/v, xylene cyanol 0.1% p/v). The mix was denatured in the thermo cycler for 5 minutes at 95 °C and then rapidly cooled down on ice. Afterwards the amplified fragments were separated by electrophoresis in 6% polyacrylamide gels with 7 M urea. Gels were visualized in the LI-COR analyzer (DNA Analyzer Gene Reader 4200, LI-COR, MWG-Biotech). They were visually scored for presence or absence of each segregating amplified band.

Initially, amplification tests were performed for all primer pairs with the parents and a subset of six progeny genotypes in order to check for amplification products of reasonable quality and adequate segregation patterns. Non segregating markers were discarded. If segregating amplification products were not of good quality, attempts were made to adjust PCR conditions for improving the amplification, if possible. Afterwards PCR reactions with suitable primers were performed with the parents and the entire mapping population.

### Data analysis

#### Mapping

For linkage mapping MAPRF software^39^,^40^ was used. Initially, only segregating parent-specific fragments were considered to generate two individual genetic maps, one from each parent.

Linkage analysis between marker fragments, estimation of recombination frequencies, and determination of linear order between linked loci including multipoint linkage analysis and the Expectation Maximization (EM) algorithm for handling missing data were performed as described^39^,^40^ using MAPRF for the computational analyses. Linked fragments were arranged into linkage groups (LG) using a minimum, commonly accepted Logarithm of the odds (LOD) threshold of 3.0 between consecutive markers. Afterwards parental LGs were projected into an integrated linkage map using individual fragments of each parent from the co dominant EST-SSR and COS markers as anchor points.

In addition, also AFLP markers common to both parents could be used efficiently as anchor points. The dosage analyses of AFLP markers common to both parents allowed distinguishing between homo and heterozygous progeny genotypes for a particular band. The estimates for RF (Recombination frequency) of such co-dominant bands have higher information values (accuracy) than those involving the same band as a dominant marker, since three instead of only two marker classes are available^40^. In order to exploit this useful feature, datasets were modified and MAPRF6 Software was adapted to handle these specific configurations. Each common fragment was converted into 2 common allelic fragments in repulsion as dominant markers. RF values were computed between both allelic and individual fragments, or between 2 pairs of allelic fragments. These estimates of higher precision were used to establish additional anchor points for parental linkage groups and to project them into the integrated map. Only anchor markers were considered which were linked with zero or had small RF (<3 cM) with individual fragments from both parents. Using this methodology, an integrated linkage map of the TKS mapping population was produced.

### Synteny study

TKS and lettuce (*Lactuca sativa*) belong to the same family (*Asteraceae*), suggesting potential synteny between both species.

For lettuce large amount of genomic resources are available which are integrated in CGPDB. Particularly an Affymetrix gene chip containing 35000 gene markers from lettuce and related species is available^41^ as well as a high-density, integrated genetic linkage map of lettuce^42^ which has nine chromosomes, one more than TKS. An ultra-high-density transcript based genetic map of lettuce comprising of 12842 unigenes was published by Truco *et al*.^43^. Mapped sequences from the mentioned *T. officinale* ESTs were blasted against the transcripts mapped on this ultra-high density lettuce genetic map.

### Functional genomics

Sequences of all EST-SSRs were blasted against a plant gene database, using Blast2Go Pro software^44^ in order to find homologous sequences. Blast hits with an e-value of 10^−10^ or less were retained. Then, mapping was performed to collect GO terms associated to blast hits. Finally, the annotation module was run in Blast2GO, in order to assign trustworthy information to each sequence.

## Additional Information

**How to cite this article**: Arias, M. *et al*. First genetic linkage map of *Taraxacum koksaghyz* Rodin based on AFLP, SSR, COS and EST-SSR markers. *Sci. Rep.* **6**, 31031; doi: 10.1038/srep31031 (2016).

## Supplementary Material

Supplementary Information

Supplementary Table S1

## Figures and Tables

**Table 1:** Characteristics of the parental and the integrated TKS linkage maps.

LG	AFLP marker			COS/SSR Loci	TM			cM
	IP1	IP2	CM					
**a) P1-L MAP**								
1	16	0	9	10	35			93.9
2	20	0	7	3	30			107.4
3	31	0	10	4	45			116.5
4	28	0	7	11	46			120.7
5	29	0	10	5	44			112.8
6	24	0	7	2	33			103.6
7	17	0	10	4	31			65.4
8	20	0	10	1	31			69.1
TOTAL Mean	185	0	70	40	295			789.4
	23.1	0.0	8.8	5.0	36.9			98.7
**b) P2-H MAP**								
1	0	16	8	10	34			80.4
2	0	16	7	0	23			83.6
3	0	29	8	5	42			119.5
4	0	33	7	10	50			122.6
5	0	27	11	5	43			114.1
6	0	25	8	1	34			123.1
7	0	18	7	8	33			84.9
8	0	24	11	3	38			81.9
TOTAL Mean	0	188	67	42	297			820.1
	0.0	23.5	8.4	5.3	37.1			102.5
**c) INTEGRATED MAP**								
						**AFLP AP**	**COS/EST-SSR AP**	**cM**
1	16	16	10	12	54	7	8	101.4
2	20	16	7	3	46	7	0	116.1
3	31	29	10	8	78	8	5	121.4
4	28	33	7	16	84	7	7	135.9
5	29	27	11	11	78	10	5	118.6
6	24	25	9	5	63	6	4	115.7
7	17	18	10	10	55	7	2	96.1
8	20	24	11	5	60	10	1	88.9
TOTAL Mean	185	188	75	70	518	62	32	894.1
	23.1	23.5	9.4	8.8	64.8	7.8	4	111.8

LG = linkage group, IP1, IP2 = Parent 1,2, CM = Common markers, TM = Total N° of markers,

AP = Anchor points based on AFLP or COS/EST-SSR markers, cM = LG in centiMorgans.; P1-L: Parent 1

low rubber producer; P2-H: Parent 2 high rubber producer.

**Table 2:** Partial Alignment of Lettuce and TKS Maps.

TC Name	TKS MAP		LettuceMap	
	LG	Position	LG	Position
TC428	1	12.8	7	933
TC128	1	36.1	6	NA
TC303	1	38.2	6	888
TC380	1	47.3	2	78
TC249	1	68.7	7	933
TC28	1	74.8	7	933
TC585	1	101.4	A^a^	1
TC454	2	33.3	7	279
TC324	3	9.7	4	190
TC494	3	27.6	4	908
TC129	3	59.8	4	1323
TC61	3	67.6	4	1323
TC80	3	68.5	4	NA
TC80	3	78.5	4	1352
TC365	4	7.9	9	154
TC295	4	12.8	7	247
TC28	4	12.9	7	933
TC1107	4	20.6	9	295
TC41A	4	50.8	2	NA
TC428	4	74	7	933
TC333	4	92.5	2	1343
TC506	5	20.5	6	352
TC42	5	23.3	6	466
TC42	5	27.1	6	NA
TC713	5	49.9	5	1401
TC713	5	49.9	5	NA
TC60	5	67.7	5	NA
TC438	5	92.5	5	155
TC77b	5	114.1	5	NA
TC461	5	118.6	6	1025
TC77	5	118.6	8	NA
TC946	6	14.1	3	703
TC946	6	71.7	3	703
TC165	6	73.7	8	1540
TC946	6	88.9	3	703
TC360	7	26.1	1	273
TC624	7	56.2	1	857
TC50A	7	61.8	1	NA
TC731	7	96.1	3	325
TC2059	8	0	A^a^	207
TC326	NM		2	104
TC366	NM		3	215

Map locations of TC Markers in Lettuce and TKS Map.

Data obtained by blasting TC markers against Lettuce Scaffolds with known

locations on the lettuce map; TC: EST-SSR markers given name; NM: not mapped;

NA: not amplified.

^a^Extra LG in the Lettuce Map.
