# Supplementary material for: First genetic linkage map of Taraxacum koksaghyz Rodin based on AFLP, SSR, COS and EST-SSR markers
Source: Sci Rep. 2016 Aug 4;6:31031. doi: 10.1038/srep31031 (PMC4973268; doi:10.1038/srep31031)
Supplement: Supplementary Information [file srep31031-s1.pdf]

Supplementary Material (Marina Arias).

First genetic linkage map of Taraxacum koksaghyz Rodin based on AFLP, SSR, COS and EST-SSR markers.

Marina Arias 1,*, Monica Hernandez 1, Naroa Remondegui1, Koen Huvenaars2, Peter van Dijk2, Enrique Ritter1.

**Supplementary Table S1**: Polymorphisms and map locations of COS, SSR and EST-SSR markers

## a) MARKERS generating segregating polymorphisms for Linkage Mapping

| Marker Name | Type | Original Name | Origin | Species | LG | Position (cM) |
|---|---|---|---|---|---|---|
| A25 | COS | A25 | 25 | *Helianthus annuus L., Lactuca sativa L* | NM | NM |
| A19 | COS | A19 | 25 | *Helianthus annuus L., Lactuca sativa L* | 8 | 43.8 |
| A27 | COS | A27 | 25 | *Helianthus annuus L., Lactuca sativa L* | 3 | 94.4 |
| B12 | COS | B12 | 25 | *Helianthus annuus L., Lactuca sativa L* | 4 | 123.1 |
| C29 | COS | C29 | 25 | *Helianthus annuus L., Lactuca sativa L* | 1 | 11.9 |
| C06 | COS | C06 | 25 | *Helianthus annuus L., Lactuca sativa L* | 6 | 27.3 |
| D05 | COS | D05 | 25 | *Helianthus annuus L., Lactuca sativa L* | 3 | 40.9 |
| SML038 | SSR | SML038 | 27 | *Lactuca sativa, Lactuca serriola* | 4 | 41.4 |
| TC1 | EST-SSR | CTOZ5807.b1_M11.ab1 | 26 | *Taraxacum officinale* | 4 | 97.2 |
| TC6 a, b | EST-SSR | CTOX13028.b1_H18.ab1 | 26 | *Taraxacum officinale* | 8, 7 | 48.9/ 38.6 |
| TC8 a, b | EST-SSR | CTOX2895.b1_M04.ab1 | 26 | *Taraxacum officinale* | 7, 5 | 39.5/ 42 |
| TC13 | EST-SSR | CTOY10260.b1_G22.ab1 | 26 | *Taraxacum officinale* | 1 | 17.7 |
| TC14 | EST-SSR | CTOX1362.b1_C06.ab1 | 26 | *Taraxacum officinale* | 7 | 22.9 |
| TC16 | EST-SSR | CTOY12295.b1_M01.ab1 | 26 | *Taraxacum officinale* | 4 | 50.7 |
| TC19 | EST-SSR | CTOY16062.b1_L08.ab1 | 26 | *Taraxacum officinale* | 8 | 26.9 |
| TC22 | EST-SSR | CTOX15864.b2_P05.ab1 | 26 | *Taraxacum officinale* | 4 | NM |
| TC25 | EST-SSR | CTOY16815.b1_N04.ab1 | 26 | *Taraxacum officinale* | 2 | 69.5 |
| TC26 | EST-SSR | CTOY6705.b1_B21.ab1 | 26 | *Taraxacum officinale* | 5 | 92.5 |
| TC27 | EST-SSR | CTOY16332.b1_G04.ab1 | 26 | *Taraxacum officinale* | 7 | 64.7 |
| TC28 a, b | EST-SSR | CTOY9502.b1_K24.ab1 | 26 | *Taraxacum officinale* | 1, 4 | 73.2/ 12.8 |
| TC29 | EST-SSR | CTOZ2149.b1_I10.ab1 | 26 | *Taraxacum officinale* | 1 | 11.5 |
| TC31 | EST-SSR | CTOY14088.b1_O18.ab1 | 26 | *Taraxacum officinale* | 5 | 9.3 |
| TC32 | EST-SSR | CTOY6345.b1_A04.ab1 | 26 | *Taraxacum officinale* | 4 | 7.9 |
| TC38 | EST-SSR | CTOX4156.b1_H08.ab1 | 26 | *Taraxacum officinale* | 4 | 133.1 |
| TC41 | EST-SSR | CTOX6656.b1_P07.ab1 | 26 | *Taraxacum officinale* | 4 | 63.3 |
| TC42 | EST-SSR | CTOX3406.b1_L12.ab1 | 26 | *Taraxacum officinale* | 5 | 27.1 |
| TC45 | EST-SSR | CTOY15765.b1_I05.ab1 | 26 | *Taraxacum officinale* | 5 | 89.8 |
| TC46 | EST-SSR | CTOX3118.b1_K11.ab1 | 26 | *Taraxacum officinale* | 6 | 7.7 |
| TC49 | EST-SSR | CTOY12359.b1_M17.ab1 | 26 | *Taraxacum officinale* | 1 | 64.8 |
| TC50 | EST-SSR | CTOY4713.b1_B03.ab1 | 26 | *Taraxacum officinale* | 7 | 59.2 |
| TC60 | EST-SSR | CTOX20584.b1_O10.ab1 | 26 | *Taraxacum officinale* | 5 | 75.3 |
| TC61 | EST-SSR | CTOX21360.b1_O12.ab1 | 26 | *Taraxacum officinale* | 3 | 65.2 |
| TC66 | EST-SSR | CTOZ5866.b1_D03.ab1 | 26 | *Taraxacum officinale* | 7 | 66.1 |
| TC69 | EST-SSR | CTOY541.b1_J15.ab1 | 26 | *Taraxacum officinale* | 2 | 33.7 |
| TC71 | EST-SSR | CTOY7144.b1_O10.ab1 | 26 | *Taraxacum officinale* | 4 | 52.7 |
| TC73 | EST-SSR | CTOZ6139.b1_F24.ab1 | 26 | *Taraxacum officinale* | 6 | 20.0 |
| TC77 | EST-SSR | CTOY6581.b1_I13.ab1 | 26 | *Taraxacum officinale* | 5 | 118.6 |
| TC80 | EST-SSR | CTOX3167.b1_M23.ab1 | 26 | *Taraxacum officinale* | 3 | 68.5 |
| TC82 | EST-SSR | CTOX10820.b1_G17.ab1 | 26 | *Taraxacum officinale* | 4 | 29.3/4.1 |
| TC83 | EST-SSR | CTOY2493.b1_J23.ab1 | 26 | *Taraxacum officinale* | 3 | 59.8 |
| TC84 | EST-SSR | CTOX21042.b2_D06.ab1 | 26 | *Taraxacum officinale* | 1 | 82.1 |
| TC333 | EST-SSR | CTOZ557.b1_J19.ab1 | 26 | *Taraxacum officinale* | 4 | 92.5 |
| TC366 | EST-SSR | CTOX2226.b1_D06.ab1 | 26 | *Taraxacum officinale* | NM | NM |
| TC129 | EST-SSR | CTOX3546.b1_C23.ab1 | 26 | *Taraxacum officinale* | 3 | 59.8 |
| TC324 | EST-SSR | CTOY9549.b1_J12.ab1 | 26 | *Taraxacum officinale* | 3 | 9.7 |
| TC128 | EST-SSR | CTOX3025.b1_B14.ab1 | 26 | *Taraxacum officinale* | 1 | 36.1 |
| TC249 | EST-SSR | CTOY7335.b1_M09.ab1 | 26 | *Taraxacum officinale* | 1 | 68.7 |
| TC295 | EST-SSR | CTOY11596.b1_G19.ab1 | 26 | *Taraxacum officinale* | 4 | 12.8 |
| TC165 | EST-SSR | CTOX11102.b1_L16.ab1 | 26 | *Taraxacum officinale* | 6 | 73.7 |
| TC585 | EST-SSR | CTOY13605.b1_J17.ab1 | 26 | *Taraxacum officinale* | 1 | 101.4 |
| TC438 | EST-SSR | CTOY8370.b1_D06.ab1 | 26 | *Taraxacum officinale* | 5 | 92.5 |
| TC713 | EST-SSR | CTOX10577.b1_A06.ab1 | 26 | *Taraxacum officinale* | 5 | 49.9 |
| TC461 | EST-SSR | CTOX11812.b1_H02.ab1 | 26 | *Taraxacum officinale* | 5 | 118.6 |
| TC2059 | EST-SSR | CTOX13056.b1_P24.ab1 | 26 | *Taraxacum officinale* | 8 | 0.0 |
| TC303 | EST-SSR | CTOY15535.b1_N19.ab1 | 26 | *Taraxacum officinale* | 1 | 38.2 |
| TC380 | EST-SSR | CTOY10164.b1_H21.ab1 | 26 | *Taraxacum officinale* | 1 | 47.3 |
| TC428 a, b | EST-SSR | CTOY6406.b1_K18.ab1 | 26 | *Taraxacum officinale* | 1, 4 | 74.0/ 12.8 |
| TC454 | EST-SSR | CTOX10662.b1_L02.ab1 | 26 | *Taraxacum officinale* | 2 | 33.3 |
| TC494 | EST-SSR | CTOX17932.b1_G20.ab1 | 26 | *Taraxacum officinale* | 3 | 27.6 |
| TC506 | EST-SSR | CTOX20035.b1_E17.ab1 | 26 | *Taraxacum officinale* | 5 | 20.5 |
| TC624 | EST-SSR | CTOY6017.b1_A18.ab1 | 26 | *Taraxacum officinale* | 7 | 56.2 |
| TC731 | EST-SSR | CTOX1290.b1_D11.ab1 | 26 | *Taraxacum officinale* | 7 | 96.1 |
| TC946 a, b, c | EST-SSR | CTOY13679.b1_M12.ab1 | 26 | *Taraxacum officinale* | 8, 7, 6 | 88.9/ 14.1/ 71.7 |
| TC1107 | EST-SSR | CTOX12029.b1_J07.ab1 | 26 | *Taraxacum officinale* | 4 | 20.6 |
| TC360 | EST-SSR | CTOX20447.b1_M23.ab1 | 26 | *Taraxacum officinale* | 7 | 26.1 |
| TC365 | EST-SSR | CTOX22195.b1_F06.ab1 | 26 | *Taraxacum officinale* | 4 | 7.9 |
| R1210L3 (1) | STS | R1210L3 (1) | 29 | *Cichorium intybus* | ---- | |
| L101L1/R1 (3) | STS | L101L1/R1 (3) | 29 | *Cichorium intybus* | ---- | |
| AF497999L2 (1) | STS | AF497999L2 (1) | 29 | *Cichorium intybus* | ---- | |

**Legend:** NM: Not Mapped; COS: Conserved Ortholog Set; SSR: Simple Sequence Repeat; EST-SSR: Expresed Sequence Tag-Simple Sequence Repeat; STS: Sequence-Tagged Sites; TC: EST-SSR given name; Origin: Bibliographic reference.

## b) MARKERS without useful amplification products or monomorphic

| Marker | Type | Original Name | Origin | Species | Marker | Type | Original Name | Origin | Species |
|---|---|---|---|---|---|---|---|---|---|
| D22 | COS | D22 | 25 | *H. annuus L., L. sativa L.* | TC11 | EST-SSR | CTOX13045.b1_J22.ab1 | 26 | *Taraxacum officinale* |
| A28 | COS | A28 | 25 | *H. annuus L., L. sativa L.* | TC12 | EST-SSR | CTOY8308.b1_G14.ab1 | 26 | *Taraxacum officinale* |
| A39 | COS | A39 | 25 | *H. annuus L., L. sativa L.* | TC15 | EST-SSR | CTOY10102.b1_L05.ab1 | 26 | *Taraxacum officinale* |
| B07 | COS | B07 | 25 | *H. annuus L., L. sativa L.* | TC17 | EST-SSR | CTOY17001.b1_B03.ab1 | 26 | *Taraxacum officinale* |
| B27 | COS | B27 | 25 | *H. annuus L., L. sativa L.* | TC18 | EST-SSR | CTOY8178.b1_D05.ab1 | 26 | *Taraxacum officinale* |
| C32 | COS | C32 | 25 | *H. annuus L., L. sativa L.* | TC20 | EST-SSR | CTOZ5155.b1_F17.ab1 | 26 | *Taraxacum officinale* |
| D22 | COS | D22 | 25 | *H. annuus L., L. sativa L.* | TC21 | EST-SSR | CTOY7019.b1_F03.ab1 | 26 | *Taraxacum officinale* |
| A33 | COS | A33 | 25 | *H. annuus L., L. sativa L.* | TC23 | EST-SSR | CTOX17822.b1_L15.ab1 | 26 | *Taraxacum officinale* |
| C01 | COS | C01 | 25 | *H. annuus L., L. sativa L.* | TC24 | EST-SSR | CTOY11228.b1_G23.ab1 | 26 | *Taraxacum officinale* |
| A01 | COS | A01 | 25 | *H. annuus L., L. sativa L.* | TC30 | EST-SSR | CTOX5018.b1_C07.ab1 | 26 | *Taraxacum officinale* |
| A02 | COS | A02 | 25 | *H. annuus L., L. sativa L.* | TC33 | EST-SSR | CTOX1131.b1_F20.ab1 | 26 | *Taraxacum officinale* |
| A03 | COS | A03 | 25 | *H. annuus L., L. sativa L.* | TC34 | EST-SSR | CTOY16350.b1_K08.ab1 | 26 | *Taraxacum officinale* |
| A04 | COS | A04 | 25 | *H. annuus L., L. sativa L.* | TC35 | EST-SSR | CTOY9350.b1_L09.ab1 | 26 | *Taraxacum officinale* |
| A05 | COS | A05 | 25 | *H. annuus L., L. sativa L.* | TC36 | EST-SSR | CTOX18095.b1_M11.ab1 | 26 | *Taraxacum officinale* |
| A06 | COS | A06 | 25 | *H. annuus L., L. sativa L.* | TC37 | EST-SSR | CTOX22367.b1_M23.ab1 | 26 | *Taraxacum officinale* |
| A07 | COS | A07 | 25 | *H. annuus L., L. sativa L.* | TC39 | EST-SSR | CTOY12186.b1_C24.ab1 | 26 | *Taraxacum officinale* |
| A08 | COS | A08 | 25 | *H. annuus L., L. sativa L.* | TC40 | EST-SSR | CTOY4029.b1_J23.ab1 | 26 | *Taraxacum officinale* |
| A09 | COS | A09 | 25 | *H. annuus L., L. sativa L.* | TC43 | EST-SSR | CTOY15841.b1_B01.ab1 | 26 | *Taraxacum officinale* |
| A10 | COS | A10 | 25 | *H. annuus L., L. sativa L.* | TC44 | EST-SSR | CTOX10565.b1_I02.ab1 | 26 | *Taraxacum officinale* |
| SML023 | SSR | SML023 | 27 | *L. serriola L., L. sativa L.* | TC47 | EST-SSR | CTOY14348.b1_H11.ab1 | 26 | *Taraxacum officinale* |
| SML026 | SSR | SML026 | 27 | *L. serriola L., L. sativa L.* | TC48 | EST-SSR | CTOY10472.b1_P01.ab1 | 26 | *Taraxacum officinale* |
| SML027 | SSR | SML027 | 27 | *L. serriola L., L. sativa L.* | TC51 | EST-SSR | CTOX16123.b2_F24.ab1 | 26 | *Taraxacum officinale* |
| SML031 | SSR | SML031 | 27 | *L. serriola L., L. sativa L.* | TC52 | EST-SSR | CTOY15494.b1_L09.ab1 | 26 | *Taraxacum officinale* |
| SML033 | SSR | SML033 | 27 | *L. serriola L., L. sativa L.* | TC53 | EST-SSR | CTOY4904.b1_P02.ab1 | 26 | *Taraxacum officinale* |
| SML034 | SSR | SML034 | 27 | *L. serriola L., L. sativa L.* | TC54 | EST-SSR | CTOX13472.b1_O07.ab1 | 26 | *Taraxacum officinale* |
| SML037 | SSR | SML037 | 27 | *L. serriola L., L. sativa L.* | TC55 | EST-SSR | CTOX844.b1_G19.ab1 | 26 | *Taraxacum officinale* |
| SML044 | SSR | SML044 | 27 | *L. serriola L., L. sativa L.* | TC56 | EST-SSR | CTOY2219.b1_F04.ab1 | 26 | *Taraxacum officinale* |
| SML054 | SSR | SML054 | 27 | *L. serriola L., L. sativa L.* | TC57 | EST-SSR | CTOY9300.b1_G21.ab1 | 26 | *Taraxacum officinale* |
| AF498000L2/R2 | STSs | AF498000L2/R2 | 29 | *Cichorium intybus* | TC58 | EST-SSR | CTOX12095.b1_N23.ab1 | 26 | *Taraxacum officinale* |
| CIFRUCTOSL3/R2 | STSs | CIFRUCTOSL3/R2 | 29 | *Cichorium intybus* | TC59 | EST-SSR | CTOX17553.b1_A22.ab1 | 26 | *Taraxacum officinale* |
| SKL95UTR9LI9R6 | STSs | SKL95UTR9LI9R6 | 29 | *Cichorium intybus* | TC62 | EST-SSR | CTOY4099.b1_E18.ab1 | 26 | *Taraxacum officinale* |
| 3262L1/R1 | STSs | 3262L1/R1 | 29 | *Cichorium intybus* | TC63 | EST-SSR | CTOY8251.b1_F23.ab1 | 26 | *Taraxacum officinale* |
| cAMP2L2/R2 | STSs | cAMP2L2/R2 | 29 | *Cichorium intybus* | TC64 | EST-SSR | CTOX10454.b1_K21.ab1 | 26 | *Taraxacum officinale* |
| CIPinvert L2/R2 | STSs | CIPinvert L2/R2 | 29 | *Cichorium intybus* | TC65 | EST-SSR | CTOY13783.b1_N14.ab1 | 26 | *Taraxacum officinale* |
| TubulinL2/R2 | STSs | TubulinL2/R2 | 29 | *Cichorium intybus* | TC67 | EST-SSR | CTOY5074.b1_C21.ab1 | 26 | *Taraxacum officinale* |
| ORS543 | SSR | ORS543 | 28 | *Helianthus annuus L* | TC68 | EST-SSR | CTOX1070.b1_L04.ab1 | 26 | *Taraxacum officinale* |
| ORS342 | SSR | ORS342 | 28 | *Helianthus annuus L* | TC70 | EST-SSR | CTOX21376.b1_O16.ab1 | 26 | *Taraxacum officinale* |
| ORS665 | SSR | ORS665 | 28 | *Helianthus annuus L* | TC72 | EST-SSR | CTOY9753.b1_B15.ab1 | 26 | *Taraxacum officinale* |
| ORS309 | SSR | ORS309 | 28 | *Helianthus annuus L* | TC74 | EST-SSR | CTOX19900.b1_H08.ab1 | 26 | *Taraxacum officinale* |
| ORS1024 | SSR | ORS1024 | 28 | *Helianthus annuus L* | TC75 | EST-SSR | CTOY16997.b1_J01.ab1 | 26 | *Taraxacum officinale* |
| ORS483 | SSR | ORS483 | 28 | *Helianthus annuus L* | TC76 | EST-SSR | CTOY4329.b1_B03.ab1 | 26 | *Taraxacum officinale* |
| ORS1041 | SSR | ORS1041 | 28 | *Helianthus annuus L* | TC78 | EST-SSR | CTOX12491.b1_E04.ab1 | 26 | *Taraxacum officinale* |
| ORS1161 | SSR | ORS1161 | 28 | *Helianthus annuus L* | TC79 | EST-SSR | CTOX1466.b1_D08.ab1 | 26 | *Taraxacum officinale* |
| ORS844 | SSR | ORS844 | 28 | *Helianthus annuus L* | TC81 | EST-SSR | CTOY10374.b1_K01.ab1 | 26 | *Taraxacum officinale* |
| ORS437 | SSR | ORS437 | 28 | *Helianthus annuus L* | TC320 | EST-SSR | CTOY7284.b1_H22.ab1 | 26 | *Taraxacum officinale* |
| ORS1146 | SSR | ORS1146 | 28 | *Helianthus annuus L* | TC326 | EST-SSR | *CTOZ1627.b1_E23.ab1 | 26 | *Taraxacum officinale* |
| ORS778 | SSR | ORS778 | 28 | *Helianthus annuus L* | TC294 | EST-SSR | CTOY10206.b1_K08.ab1 | 26 | *Taraxacum officinale* |
| ORS1179 | SSR | ORS1179 | 28 | *Helianthus annuus L* | TC93 | EST-SSR | CTOX21614.b1_L03.ab1 | 26 | *Taraxacum officinale* |
| ORS307 | SSR | ORS307 | 28 | *Helianthus annuus L* | TC444 | EST-SSR | CTOZ1761.b1_A10.ab1 | 26 | *Taraxacum officinale* |
| ORS687 | SSR | ORS687 | 28 | *Helianthus annuus L* | TC386 | EST-SSR | CTOY11590.b1_K17.ab1 | 26 | *Taraxacum officinale* |
| ORS750 | SSR | ORS750 | 28 | *Helianthus annuus L* | TC188 | EST-SSR | CTOX13943.b1_N05.ab1 | 26 | *Taraxacum officinale* |
| ORS735 | SSR | ORS735 | 28 | *Helianthus annuus L* | TC365 | EST-SSR | CTOX22195.b1_F06.ab1 | 26 | *Taraxacum officinale* |
| Let1 | COS-EST | QGF13H13.yg.ab1 | 27 | *L. serriola L., L. sativa L.* | TC418 | EST-SSR | CTOY3757.b1_J04.ab1 | 26 | *Taraxacum officinale* |
| Let2 | COS-EST | QGD7O20.yg.ab1 | 27 | *L. serriola L., L. sativa L.* | TC1555 | EST-SSR | CTOY3131.b1_E15.ab1 | 26 | *Taraxacum officinale* |
| Let3 | COS-EST | QG_CA_Contig1410 | 27 | *L. serriola L., L. sativa L.* | TC562 | EST-SSR | CTOY10304.b1_P08.ab1 | 26 | *Taraxacum officinale* |
| Let4 | COS-EST | QG_CA_Contig6178 | 27 | *L. serriola L., L. sativa L.* | TC489 | EST-SSR | CTOX16428.b1_H04.ab1 | 26 | *Taraxacum officinale* |
| Let5 | COS-EST | QGF23G16.yg.ab1 | 27 | *L. serriola L., L. sativa L.* | TC543 | EST-SSR | CTOX4545.b1_B10.ab1 | 26 | *Taraxacum officinale* |
| TC2 | EST-SSR | CTOX19837.b1_I16.ab1 | 26 | *Taraxacum officinale* | TC624 | EST-SSR | CTOY6017.b1_A18.ab1 | 26 | *Taraxacum officinale* |
| TC3 | EST-SSR | CTOY2052.b1_H09.ab1 | 26 | *Taraxacum officinale* | TC604 | EST-SSR | CTOY1842.b1_D06.ab1 | 26 | *Taraxacum officinale* |
| TC4 | EST-SSR | CTOX15289.b1_B08.ab1 | 26 | *Taraxacum officinale* | TC946 | EST-SSR | CTOY13679.b1_M12.ab1 | 26 | *Taraxacum officinale* |
| TC5 | EST-SSR | CTOX18135.b1_M21.ab1 | 26 | *Taraxacum officinale* | TC1022 | EST-SSR | CTOY6770.b1_C14.ab1 | 26 | *Taraxacum officinale* |
| TC7 | EST-SSR | CTOX2872.b1_P21.ab1 | 26 | *Taraxacum officinale* | TC712 | EST-SSR | CTOX1043.b1_E22.ab1 | 26 | *Taraxacum officinale* |
| TC9 | EST-SSR | CTOX6666.b1_D11.ab1 | 26 | *Taraxacum officinale* | TC513 | EST-SSR | CTOX20664.b1_P06.ab1 | 26 | *Taraxacum officinale* |
| TC10 | EST-SSR | CTOX18729.b2_B04.ab1 | 26 | *Taraxacum officinale* | | | | | |

**Legend**: NM: Not Mapped; COS: Conserved Ortholog Set; SSR: Simple Sequence Repeat; EST-SSR: Expresed Sequence Tag-Simple Sequence Repeat; STS: Sequence-Tagged Sites; TC: EST-SSR given name; Reference: Bibliographic reference.

**Supplementary Table S2:** Obtained homologies of TC markers using Blast2Go.

| SEQNAME | DESCRIPTION | LENGTH | HITS | E-VALUE | SIM MEAN | GO | GO NAMES LIST | ENZYME CODES |
|---|---|---|---|---|---|---|---|---|
| TC1107 | zinc ion binding | 892 | 20 | 1.9E-85 | 69.65% | 1 | F:metal ion binding | - |
| TC128 | u-box domain-containing protein 7-like | 928 | 20 | 6.3E-33 | 61.65% | 1 | F:protein binding | - |
| TC129 | mannanendo- -beta-mannosidase 2-like | 914 | 20 | 3.9E-41 | 78.25% | 2 | F:hydrolase activity, hydrolyzing O-glycosyl compounds; P:carbohydrate metabolic process | - |
| TC13 | coiled-coil domain-containing protein 97 | 906 | 20 | 5.9E-103 | 78% | 2 | P:biological_process; C:cellular_component | - |
| TC165 | probable arabinosyltransferase arad1 | 819 | 20 | 2.3E-24 | 79.65% | 3 | P:biological_process; F:transferase activity; P:metabolic process | - |
| TC19 | protein n-lysinemethyltransferase mettl21a | 905 | 20 | 6.6E-123 | 83.25% | 4 | F:histone acetyltransferase activity; F:methyltransferase activity; P:histone acetylation; P:methylation | EC:2.3.1.48; EC:2.3.1 |
| TC2059 | actin-related protein 9 | 392 | 20 | 7.6E-45 | 83.9% | 3 | C:Ino80 complex; P:chromatin remodeling; P:vegetative to reproductive phase transition of meristem | - |
| TC22A | Something about silencing protein 10 | 906 | 20 | 4.8E-58 | 67.05% | 0 | - | - |
| TC22B | Something about silencing protein 10 | 906 | 20 | 4.8E-58 | 67.05% | | - | - |
| TC249 | Coatomer subunit zeta-1-likeprotein | 790 | 20 | 2.0E-100 | 95.65% | 8 | C:membrane; P:protein folding; P:response to heat; P:response to high light intensity; P:protein transport; P:response to endoplasmic reticulum stress; P:response to hydrogen peroxide; P:positive regulation of transcription, DNA-templated | - |
| TC25 | uncharacterized calcium-binding protein at1g02270-like isoform x2 | 883 | 20 | 9.7E-89 | 78.75% | 1 | F:calcium ion binding | - |
| TC27 | duf581 family protein | 660 | 20 | 9.0E-20 | 79.35% | | - | - |
| TC28 | gpi-anchor transamidase isoform x1 | 874 | 20 | 1.2E-80 | 96.85% | 7 | C:GPI-anchor transamidase complex; F:GPI-anchor transamidase activity; F:cysteine-type endopeptidase activity; P:proteolysis; P:sterol biosynthetic process; P:attachment of GPI anchor to protein; P:sphingoid biosynthetic process | EC:3.4.22; EC:3.4; EC:3 |
| TC29 | Nucleolar protein | 558 | 20 | 4.5E-31 | 63.45% | 3 | P:root hair cell differentiation; P:biological_process; C:integral component of membrane | - |
| TC295 | PREDICTED: uncharacterized protein LOC101202853 isoform X1 | 485 | 20 | 1.4E-16 | 68.65% | 1 | C:Golgi apparatus | - |
| TC303 | glycerol-3-phosphate acyltransferase | 818 | 20 | 3.0E-61 | 75.25% | 6 | C:chloroplast; F:transferase activity, transferring acyl groups; P:isoprenoid biosynthetic process; P:monocarboxylic acid metabolic process; P:pigment biosynthetic process; P:glycerophospholipid biosynthetic process | - |
| TC32 | regulator of nonsense transcripts upf3-like isoform x2 | 825 | 20 | 9.0E-30 | 53.05% | 1 | F:nucleotide binding | - |
| TC324 | PREDICTED: uncharacterized protein LOC104219523 | 874 | 20 | 3.5E-98 | 86.35% | 9 | P:maltose metabolic process; P:phosphatidylglycerol biosynthetic process; P:embryo development ending in seed dormancy; P:thylakoid membrane organization; P:stomatal complex morphogenesis; P:iron-sulfur cluster assembly; P:starch biosynthetic process; P:isopentenyl diphosphate biosynthetic process, methylerythritol 4-phosphate pathway; P:positive regulation of catalytic activity | - |
| TC360 | formin-like protein 8 isoform x2 | 653 | 20 | 6.8E-34 | 69.65% | 1 | P:phosphatidylinositol biosyntheticprocess | - |
| TC365 | probable sphingolipid transporter spinster homolog 2 | 873 | 20 | 1.9E-110 | 87.65% | 6 | C:intracellular; C:integral component of membrane; P:fatty acid beta-oxidation; P:sterol biosynthetic process; P:protein import into peroxisome matrix; P:sphingoid biosynthetic process | - |
| TC366 | probable inactive receptor kinase at1g48480 | 943 | 20 | 1.1E-68 | 72.75% | 4 | C:membrane; F:nucleotide binding; F:protein kinase activity; P:phosphorylation | - |
| TC38 | PREDICTED: uncharacterized protein LOC105160266 isoform X3 | 704 | 12 | 2.4E-15 | 51.83% | 1 | P:cellular process | - |
| TC41A | PREDICTED: uncharacterized protein LOC105157371 | 834 | 20 | 4.8E-81 | 91.95% | 0 | - | - |
| TC41B | PREDICTED: uncharacterized protein LOC105157371 | 834 | 20 | 4.8E-81 | 91.95% | | - | - |
| TC42 | udp-d-apioseudp-d-xylose synthase 2-like | 864 | 20 | 4.1E-172 | 95.8% | 3 | F:catalytic activity; F:coenzyme binding; P:metabolic process | - |
| TC428 | coatomersubunit zeta-1-like | 832 | 20 | 3.0E-99 | 95.05% | 8 | C:membrane; P:protein folding; P:response to heat; P:response to high light intensity; P:protein transport; P:response to endoplasmic reticulum stress; P:response to hydrogen peroxide; P:positive regulation of transcription, DNA-templated | - |
| TC438 | Unnamed protein product | 719 | 20 | 1.6E-60 | 78.1% | | - | - |
| TC45A | Uncharacterized serine-rich protein | 887 | 20 | 1.8E-23 | 79.95% | | - | - |
| TC45B | Uncharacterized serine-rich protein | 887 | 20 | 1.7E-23 | 79.95% | | - | - |
| TC461 | udp-glycosyltransferase 84b2-like | 789 | 20 | 4.1E-82 | 65.9% | 2 | F:transferase activity, transferring hexosyl groups; P:metabolic process | - |
| TC46A | polypyrimidine tract-binding protein homolog 2-like isoform x1 | 859 | 20 | 1.1E-35 | 66.5% | 1 | F:nucleotide binding | - |
| TC46B | polypyrimidine tract-binding protein homolog 2-like isoform x1 | 859 | 20 | 1.1E-35 | 66.5% | 1 | F:nucleotide binding | - |
| TC46C | polypyrimidine tract-binding protein homolog 2-like isoform x1 | 859 | 20 | 1.1E-35 | 66.5% | 1 | F:nucleotide binding | - |
| TC49 | rac-like gtp-binding protein arac7 | 899 | 20 | 9.0E-127 | 94.55% | 10 | C:intracellular; C:plasma membrane; F:GTPase activity; F:sphingomyelin phosphodiesterase activity; F:GTP binding; P:intracellular protein transport; P:nucleocytoplasmic transport; P:actin filament organization; P:small GTPase mediated signal transduction; P:metabolic process | EC:3.6.1; EC:3.1; EC:3.1.4.12; EC:3.1.1; EC:3.6.1.15 |
| TC494 | protein nrt1 ptr family –like | 846 | 20 | 7.7E-117 | 83.3% | 3 | C:membrane; F:transporter activity; P:transport | - |
| TC506 | probable inactive leucine-rich repeat receptor-like protein kinase at3g03770 | 917 | 20 | 2.0E-14 | 70.55% | 4 | F:kinase activity; P:phosphorylation; P:response to stimulus; P:organic substance metabolic process | - |
| TC50A | rab6-interacting golgin-like | 868 | 20 | 6.7E-76 | 94.5% | 2 | C:plasma membrane; P:response to auxin | - |
| TC50B | rab6-interacting golgin-like | 868 | 20 | 6.7E-76 | 94.7% | | - | - |
| TC585 | f-box family isoform 1 | 491 | 20 | 2.2E-35 | 55.8% | 2 | F:DNA binding; P:regulation of transcription, DNA-templated | - |
| TC60 | protein elf4-like 4-like | 718 | 20 | 1.5E-45 | 85.1% | | - | - |
| TC61A | mannanendo- -beta-mannosidase 2-like | 800 | 20 | 1.8E-24 | 76.9% | 2 | F:hydrolase activity, hydrolyzing O-glycosyl compounds; P:carbohydrate metabolic process | - |
| TC61B | mannanendo- -beta-mannosidase 2-like | 800 | 20 | 1.8E-24 | 76.9% | 2 | F:hydrolase activity, hydrolyzing O-glycosyl compounds; P:carbohydrate metabolic process | - |
| TC61C | mannanendo- -beta-mannosidase 2-like | 800 | 20 | 1.8E-24 | 76.9% | 2 | F:hydrolase activity, hydrolyzing O-glycosyl compounds; P:carbohydrate metabolic process | - |
| TC624 | benzyl alcohol o-benzoyltransferase | 918 | 20 | 0.0E0 | 86.55% | 2 | F:transferase activity, transferring acyl groups other than amino-acyl groups; P:metabolic process | - |
| TC66 | duf581 family protein | 683 | 20 | 7.1E-21 | 76.4% | | - | - |
| TC69 | rho gdp-dissociation inhibitor 1 | 826 | 20 | 3.4E-65 | 75.75% | 3 | C:cytoplasm; F:Rho GDP-dissociation inhibitor activity; P:regulation of catalytic activity | - |
| TC71 | novel plantsnare 13-like | 871 | 20 | 3.0E-93 | 90.6% | 0 | - | - |
| TC713 | rho gtpase activation protein with ph domain isoform 1 | 857 | 20 | 6.3E-61 | 71.5% | 1 | P:signal transduction | - |
| TC73 | udp-glucuronic acid decarboxylase 6-like | 569 | 20 | 1.9E-56 | 89.7% | 3 | F:catalytic activity; F:coenzyme binding; P:metabolic process | - |
| TC731 | ---NA--- | 269 | 0 | - | - | | - | - |
| TC77 | Pyridoxal biosynthesis protein | 876 | 20 | 6.2E-159 | 97.05% | 18 | C:cytosol; C:plasma membrane; C:endomembrane system; F:pyridoxal 5'-phosphate synthase (glutamine hydrolysing) activity; F:protein homodimerization activity; F:protein heterodimerization activity; P:maltose metabolic process; P:pentose-phosphate shunt; P:cellular amino acid metabolic process; P:response to lipid hydroperoxide; P:pyridoxine biosynthetic process; P:response to UV-B; P:response to non-ionic osmotic stress; P:chlorophyll metabolic process; P:starch biosynthetic process; P:hyperosmotic salinity response; P:pyridoxal phosphate biosynthetic process; P:positive regulation of catalytic activity | EC:4.3.3.6 |
| TC80A | serine threonine-protein kinase d6pk | 915 | 20 | 2.0E-92 | 88.2% | 7 | C:nucleolus; C:cytoplasm; F:protein kinase activity; F:ATP binding; P:microtubule cytoskeleton organization; P:cytokinesis by cell plate formation; P:protein phosphorylation | - |
| TC80B | serine threonine-protein kinase d6pk | 915 | 20 | 2.0E-92 | 88.2% | 7 | C:nucleolus; C:cytoplasm; F:protein kinase activity; F:ATP binding; P:microtubule cytoskeleton organization; P:cytokinesis by cell plate formation; P:protein phosphorylation | - |
| TC82A | tpr repeat-containing thioredoxin ttl1-like | 777 | 20 | 1.1E-16 | 59.2% | 2 | P:cell redox homeostasis; C:cell | - |
| TC82B | tpr repeat-containing thioredoxin ttl1-like | 777 | 20 | 1.1E-16 | 59.2% | 2 | P:cell redox homeostasis; C:cell | - |
| TC83A | protein elf4-like 4 | 734 | 20 | 2.6E-54 | 89.85% | | - | - |
| TC83B | protein elf4-like 4 | 734 | 20 | 2.6E-54 | 89.85% | | - | - |
| TC83C | protein elf4-like 4 | 734 | 20 | 2.6E-54 | 89.85% | | - | - |
| TC84A | auxilin-related protein 2 isoform x2 | 712 | 20 | 5.3E-16 | 90.15% | 2 | F:transferase activity; P:metabolic process | - |
| TC84B | auxilin-related protein 2 isoform x2 | 712 | 20 | 5.3E-16 | 90.15% | 2 | F:transferase activity; P:metabolic process | - |
| TC84C | auxilin-related protein 2 isoform x2 | 712 | 20 | 5.3E-16 | 90.15% | 2 | F:transferase activity; P:metabolic process | - |
| TC84D | auxilin-related protein 2 isoform x2 | 712 | 20 | 5.3E-16 | 90.15% | 2 | F:transferase activity; P:metabolic process | - |

**Legend:** F: Molecular Function; P: Biological Process; C: Cellular Component; GO: Gene Ontology; SIM Mean: % of similitude; Seq Name: Sequence Name.
